# A short history of sarcopenia and frailty and their impact on advanced chronic liver disease

**DOI:** 10.25122/jml-2024-0304

**Published:** 2024-07

**Authors:** Denisa Cuciureanu, Petruta-Violeta Filip, Corina-Silvia Pop, Sorina-Laura Diaconu

**Affiliations:** 1 Carol Davila University of Medicine and Pharmacy, Bucharest, Romania; 2 Department of Internal Medicine II and Gastroenterology, Emergency University Hospital of Bucharest, Romania

**Keywords:** frailty, sarcopenia, malnutrition, advanced chronic liver disease, health care costs, history

## Abstract

Sarcopenia, first introduced as a concept by I. Rosenberg in 1989, has since been extensively studied, particularly in its correlation with chronic diseases. In recent years, sarcopenia has been increasingly associated with advanced chronic liver disease, leading to a lower quality of life and poor outcomes for these patients. Studies have shown that sarcopenia has a prevalence of 33% in individuals with advanced chronic liver disease, impacting not only the patient’s health but also contributing to increased healthcare costs. The prevalence of frailty in patients with advanced chronic liver disease is 27%. Given the high prevalence of sarcopenia and frailty in this population, early diagnosis and treatment are crucial to improving patient quality of life outcomes and reducing the strain on healthcare systems globally.

## INTRODUCTION

The world population is increasing rapidly, with the older population (over 65 years) expected to reach 3.1 billion by 2100. Advances in the medical field also extend life expectancy, leading to an anticipated rise in the population aged 80 and over to 0.9 billion by 2100 [1,2].

With aging come various risks and health problems, including chronic disease, frailty, and sarcopenia, which increase the health needs of older persons and the financial burden on health systems. Age-related ailments are often interconnected, and their progression can exacerbate one another. Physical activity is one of the important measures that can be taken to prevent or at least delay the onset of these diseases [3]. Research has confirmed that physical activity is essential for healthy aging [4]. It lowers the risk of several chronic conditions, such as obesity, hypertension, type 2 diabetes, coronary heart disease, and high cholesterol [4–7]. The most significant and direct impact that physical activity has is on the quantity and quality of muscle mass [8]. On average, individuals lose about 1% of muscle mass per year until age 70, after which the loss accelerates to approximately 1.5% per year. Nevertheless, patients with advanced chronic liver disease lose up to 3% per year, increasing their risk of mortality, regardless of their liver disease severity [8]. This directly relates to sarcopenia and frailty. Sarcopenia is a common characteristic in patients with advanced chronic liver disease and a compelling risk factor for general mortality. While frailty and sarcopenia were once considered synonymous, it is now recognized that sarcopenia is a key component of frailty. Frailty syndrome is defined by a reduced physiological reserve and a raised susceptibility to health stressors that lead to poor health outcomes [9]. Nevertheless, when did we first begin to discuss them, and how can the early detection of these two intertwined syndromes reduce healthcare costs and, most of all, improve the quality of life for older patients?

### Aim

This study aimed to investigate the historical development of the terms 'frailty’ and 'sarcopenia’ in medical literature to better understand their evolution and impact on patients’ quality of life, outcomes, and the strain they place on healthcare systems. Moreover, we aimed to identify the prevalence of frailty and sarcopenia in patients with advanced chronic liver disease. This knowledge will help us better screen these patients, improve their quality of life, and reduce complications and hospitalizations, particularly as we see a growing number of younger patients admitted with advanced chronic liver disease, contrary to expectations that this population would primarily consist of older individuals.

## MATERIAL AND METHODS

Very few articles mention the history of both frailty and sarcopenia, and we wanted to search the literature to trace the beginning and evolution of these two syndromes because what better way to understand a certain syndrome or disease than going straight to the origin? Additionally, the prevalence of frailty and sarcopenia among patients with advanced chronic liver disease shows significant variation, prompting us to investigate the median prevalence rates and what to expect when screening.

We divided our search into two parts: the first focused on the historical background of frailty and sarcopenia, while the second examined their prevalence in patients with advanced chronic liver disease, considering that liver cirrhosis has become one of the major causes of global health loss, although it remains one of the most preventable one [10].

We conducted our search in the PubMed database, first searching about the history of frailty and sarcopenia, using the terms frailty, sarcopenia, history, and first mention. The earliest article we found discussing frailty was published in 1968 by T.D. O’Brien, while the first mention of sarcopenia was in 1989 by Irwin Rosenberg. Because we were interested in the evolution and development of frailty and sarcopenia, we did not use any time-related restrictions for this search. We included articles that mentioned sarcopenia, frailty, muscle function, nutrition, and sarcopenia/frailty-related impairments/diseases.

For the second part of our research, we restricted our search to articles published from 2019 onwards, using the additional search terms advanced chronic liver disease, cirrhosis, and prevalence. We included articles that defined sarcopenia or frailty, described methods or tools for diagnosing these syndromes, and studies or reviews that covered their prevalence. We also included the World Health Organization brochure and the European Commission aging report. For the second part of our search, we included only articles that were considered related to our interest by at least two reviewers.

### Sarcopenia

The term *sarcopenia* is derived from Greek, from the words sarx (flesh) and penia (loss) [11,12]. Irwin Rosenberg first introduced it in 1989, following the Third National Health and Nutrition Examination Survey. He proposed both 'sarcopenia' and 'sarcomalacia’ as terms for the decrease of lean body mass in order to get more attention on the subject [13]. He believed that there is a strong correlation between sarcopenia and physical behavior in older patients and even mentioned the 'frail elderly’ and the wonderful capacity of muscle function recovery [13].

In 1994, at the Workshop of Sarcopenia, William J. Evans emphasized that the most significant change in body composition among aging individuals is the loss of skeletal muscle mass [14]. He also mentioned that muscle reduction in the elderly is a direct cause of muscle strength decrease and that not all muscle mass loss is associated with muscle quality [14].

**Figure 1:**
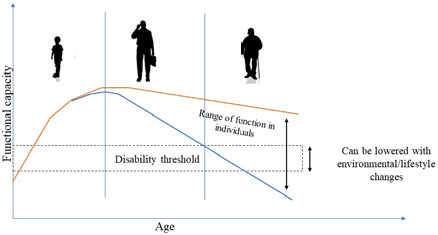
Range of function in adults - adapted from World Health Organization (WHO) diagram. Geneva: WHO; 2000.

In 2000, the World Health Organization (WHO) recognized sarcopenia as a significant risk factor for loss of independence and various morbidities in the elderly [15,16]. It targeted sarcopenia as modifiable with lifestyle changes (Figure 1) [16,17].

**Figure 2:**
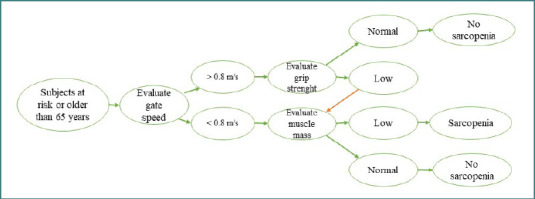
Algorithm for finding cases of sarcopenia in individuals – adapted after EWGSOP suggested algorithm

In 2010, the European Working Group on Sarcopenia in Older People (EWGSOP) published a definition of sarcopenia that has since been adopted worldwide [18]. The definition meant better and more accessible care for patients with sarcopenia and those at risk. Given that in October 2016, sarcopenia was recognized as a distinct condition, gaining an ICD-10-C code established by the Centers for Disease Control and Prevention (CDC), and because an update seemed justified by the scientific evidence accumulated, there was a new meeting of the EWGSOP [19]. This update introduced tools and explicit criteria for identifying sarcopenia in both research populations and clinical practice (Figure 2). These tools include the evaluation of function - grip strength, performance - gate speed, and quantity - muscle mass [18,20–22].

The increasing burden on healthcare systems makes it essential for clinical practitioners to screen early for sarcopenia to prevent, delay, and treat the condition and, in some cases, even reverse it with effective interventions. This is the likely path to increase the quality of life in the elderly and reduce the on-growing cost for healthcare systems worldwide [23,24].

Today, many of the epidemiological and pathophysiological characteristics of sarcopenia are better acknowledged than they were 10-20 years ago [18].

Initially, sarcopenia was thought to be solely linked to older adults and aging, but it is now known that the condition can begin in early adulthood [18,25]. Multiple factors contribute to the development of sarcopenia [25,26]. Furthermore, it has become easier to recognize sarcopenia in clinical practice, as it is now understood to be primarily a disease of muscle strength (with low muscle strength) rather than simply a loss of muscle mass [27,28].

To provide optimal care for patients with sarcopenia, improve their quality of life, and reduce the burden on healthcare systems (as sarcopenia is a costly disease), healthcare professionals must rapidly identify the areas where sarcopenia has had the most impact [29]. So, what are the areas most affected by sarcopenia?

1. Independence: sarcopenia increases the risk of falls and/or fractures [28] mobility disorders [20], and reduces the ability to perform daily living activities [30].
2. Multiple organs: Sarcopenia affects the heart (cardiac disease), lungs (respiratory disease), brain (cognitive impairment), liver (advanced liver disease), and gut (inflammatory bowel disease) [18,31–35].

These issues lead to poor quality of life, the need for nursing home placement, and, in some cases, even death [3,36,37]. A 2015 study by Sousa and colleagues revealed that the hospitalization costs of patients with sarcopenia were, on average, €1000 higher per patient (regardless of age) than for patients without sarcopenia [23].

**Figure 3:**
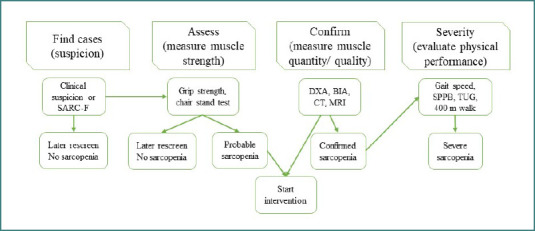
Algorithm for identifying sarcopenia cases, as suggested by EWGSOP2, adapted from Cruz AJ et al. [18]. SARC-F, questionnaire, and score for the assessment of sarcopenia; DXA, dual-energy x-ray absorptiometry; BIA, bioelectrical impedance analysis; CT, computed tomography; MRI, magnetic resonance imaging; SPPB, Short Physical Performance Battery test; TUG, Timed Up and Go test

So, what can we do? We use screening tools! (Figure 3)[18].

### Frailty

The concept of frailty first emerged in 1968 in a study conducted by O’Brien and colleagues, who used the term 'frail and elderly’ to describe a heterogeneous group of individuals. It was a yearlong study involving 48 persons meant to evaluate the problems related to ‘frail and elderly’ [38,39]. The first formal definition of frailty appeared in 1988, when Winograd and colleagues conducted a study on patients over 65, identifying that frail adults commonly shared between one and fifteen geriatric conditions and experienced longer hospital stays [39,40]. In 2001, Fried and colleagues provided a more structured definition, characterizing frailty as a clinical syndrome based on the presence of three or more of five criteria, which they identified in their Cardiovascular Health Study [41]. It was recognized in 2008 that frail is not a synonym of disability or comorbidity but a clinical syndrome and a pre-disability state, although a consensus definition or a common assessment tool was not reached, as highlighted by The Frailty Task Force of Geriatric Advisory Panel) [42-44]. It is believed that one of the most important roles in the frailty pathogenesis that occurs with old age is played by sarcopenia. It is known that progressive muscle wasting happens with aging. This is very important because physical impairment leads to disability, and this is one of the major causes of hospitalizations or institutionalization in nursing homes for elderly patients – and this means costs of billions of euros for healthcare systems [45]. Furthermore, the loss of lean body mass increases the risk of obesity, as decreased energy expenditure can lead to fat accumulation, which in turn exacerbates health impairments and comorbidities [45].

### Advanced chronic liver disease

Advanced chronic liver disease is responsible for over two million deaths annually worldwide [10,46]. It also causes a significant financial burden on the healthcare system and great disability to patients, affecting their quality of life. The true scale of the problem may be underestimated due to insufficient data in certain regions [46].

Three key entities—sarcopenia, malnutrition, and frailty—have clinical relevance in advanced chronic liver disease. What roles do these factors play, and how can they be addressed to improve quality of life, prevent disability, and reduce healthcare costs?

**Sarcopenia** in advanced chronic liver disease has been increasingly studied in recent years and is now characterized by the progressive and diffuse loss of skeletal muscle mass, function, and strength [47-49]. A recent meta-analysis, which was dominated by male participants, found that the overall prevalence of sarcopenia in patients with advanced chronic liver disease was 33%. The prevalence varied based on Child-Pugh (CP) classification, with 33% in CP-A, 36% in CP-B, and 46% in CP-C patients. This means that 1 in 3 patients with advanced chronic liver disease also suffers from sarcopenia [50]. This leads to the evolution of multiple complications, such as ascites, infections, or hepatic encephalopathy, which translates to an inferior quality of life, poor survival (especially for sarcopenic patients awaiting liver transplants), and longer hospitalization days with higher costs [50-52]. While malnutrition contributes to sarcopenia, the two are not equivalent, as sarcopenia can arise from various other conditions [47,53].

**Malnutrition** is represented by a defined change in mental and physical functions consequent to a modified body composition and cell quantity, which leads to poor quality of life and scarce clinical outcomes [47,48]. According to the Global Leadership Initiative on Malnutrition, since 2016, the diagnosis of malnutrition necessitates etiological and phenotypical criteria (reduced food intake and/or absorption in the presence of inflammation from acute/chronic disease and a reduced muscle mass/body mass index or weight) [53]. Among patients with advanced liver disease, malnutrition is linked to a higher risk of complications, extended hospital stays, and increased mortality [54]. Although malnutrition is relatively easy to assess in older patients, it can be difficult to evaluate in those with advanced liver disease due to low transferrin and albumin levels. Furthermore, in cases of fluid retention, weight and body mass index (BMI) become unreliable metrics [47,55]. This means a nutritional evaluation should be implemented in all medical centers, especially gastroenterology wards, along with specific diagnostic criteria for these patients [56]. The prevalence of malnutrition varies greatly, between 5-92% in patients with advanced chronic liver disease, denoting the need for accurate tools and specialized personnel to assess it.

**Frailty**, originally a geriatric concept, is defined as a state of physiological decline that leads to increased vulnerability and diminished reserve capacity. It was first developed to identify elderly individuals at higher risk for falls, disability, dependency, worse health outcomes, hospitalization, nursing home admission, and death [47,48]. Assessing physical, functional, and cognitive abilities is at the base of frailty. This makes sarcopenia and malnutrition significant parts of it. The link between frailty and advanced chronic liver disease has been extensively studied, with evidence showing a significant association between frailty and mortality in patients awaiting liver transplants, particularly when assessed using the six-minute walk test [57]. In a systematic review from 2024, Xie and colleagues concluded that the overall prevalence of frailty in patients with advanced chronic liver disease was 27%. They determined that frailty has a high prevalence in these patients and that compared with non-frail patients, these tend to be older, male, with reduced BMI and worse liver function [58].

## CONCLUSION

Although sarcopenia and frailty are relatively new concepts in the context of advanced chronic liver disease, the increasing number of studies highlights their significant prevalence (27% for frailty and 33% for sarcopenia). We need to find the optimal tools that medical personnel can easily use in clinical practice to identify malnutrition, sarcopenia, and frailty in patients with advanced chronic liver disease in order to improve the quality of life, health outcomes, decrease morbidity and mortality, and equally important to reduce medical expenses and the burden it has on the health care system.
